# Supplementary material for: Effects of joint mobilization combined with acupuncture on pain, physical function, and depression in stroke patients with chronic neuropathic pain: A randomized controlled trial
Source: PLoS One. 2023 Aug 24;18(8):e0281968. doi: 10.1371/journal.pone.0281968 (PMC10449141; doi:10.1371/journal.pone.0281968)
Supplement: S1 File — (PDF) [file pone.0281968.s002.pdf]

연구계획서(인간대상연구용)

Version 2.0

연구제목: 한방 치료를 병행한 관절 가동 운동이 뇌졸중 환자의 통증, 우울 및 신체 기능에 미치는 효과

단국대학교 일반대학원 운동의과학과 이호성

1. 연구의 목적 및 배경

• 뇌졸중은 성인 상지 (UL) 운동 장애의 가장 흔한 원인이며 (Maciejasz P, 2014), 편마비성 어깨 통증 (HSP)은 생존자의 약 30%가 1년 이내에 경험하는 심각한 합병증이다 (Adey-Wakeling, 2015).

• 뇌졸중 환자의 3분의 1만이 처음 6개월 이내에 기능을 회복하고 운동 회복이 빈약하거나 불충분하다 (Dobkin, 2005). 뇌졸중 환자의 45 % 미만이 완전한 기능 회복을 달성할 가능성이 있는 반면, 대부분은 다양한 종류의 후유 장애와 일상 생활 활동 수행의 어려움이 나타난다 (Kelly-Hayes et al., 2003; Marini et al. , 2004; Flynn et al., 2008).

• 뇌졸중은 운동 제어 및 감각 결핍 외에도 통증, 경련, 관절 제약, 피부 또는 혈관 손상과 같은 일반적인 합병증이 나타나며 이는 뇌졸중 관리에서 가장 중요한 문제이다 (Lang et al., 2013; Ver et al., 2015).

• 보행 기능의 회복은 재활의 주요 목표이며, 대부분의 뇌졸중 환자는 보행 속도 감소로 보행의 어려움이 나타난다 (Yoo & Chung, 2015). 독립적인 보행은 뇌졸중 후 일상 생활 활동을 수행하는 데 중요한 요소이며, 선행연구에 따르면 감각 운동 기능 장애는 관절 운동 범위 (ROM)의 제한과 마비측의 하지 근육 약화를 유발하여 앉아서 일어 서기 및 보행과 같은 기능적 활동을 수행하는 데 어려움을 겪는다 (Lyn et al. al., 2006; Fujita et al., 2011).

• 따라서 뇌졸중 환자의 신체 기능을 향상시키기 위해서는 관절의 통증을 줄이고 운동 범위를 넓히는 것이 필요하다고 생각된다. 이러한 문제를 해결하고 상지 및 하지 기능을 회복 및 개선하는데 도움을 주고 재활을 제공하도록 설계된 다양한 방법이 치료사로부터 많이 사용되고 있다.

• 멀리건은 관절 가동 (JM) 기법으로 움직임을 이용한 가동을 처음으로 제안했습니다. 관절 가동은 운동의 3 단계와 볼록 오목 규칙을 통해 통증을 완화하고 움직임을 개선하며 구축을 치료하는 기계적 효과에 사용되는 정형 외과적 방법이다 (Kaltenborn et al., 2007).

• 관절 가동은 견인, 압박 및 활주를 포함하며 I-III 등급으로 나누어진다. 등급 I은 접합 표면을 분리하지 않고 약간의 강도로 신연을 시행한다. 등급 II는 관절낭 내에서 관절 주위 조직을 늘려 관절 표면을 분리한다. III 등급은 관절낭이 충분히 늘어날 수 있도록 충분한 신연 또는 주를 시행한다 (Kaltenborn et al., 2007). 볼록하고 오목한 규칙에 따르면 볼록한 관절 표면을 활주하는 것은 뼈의 이동 방향과 반대 방향으로 활주하는 것을 의미한다. 오목한 관절 표면을 활주하는 것은 뼈의 이동 방향과 반대 방향으로 미끄러짐을 시행하는 것이다. 오목한 관절 표면을 분리할 때 뼈의 긴 축을 따라 당겨진다. 그러나 볼록한 관절 표면을 신연할 때는 관절 표면을 분리해야한다 (Kaltenborn et al., 2007).

• 관절 운동은 관절 부위의 직접적인 움직임에 의해 구축을 방해하는 기계적 효과뿐만 아니라 관절 수용체를 촉진하여 관련 근육의 억제 또는 촉진을 촉진하는 관절 운동 반사 효과도 있다 (Ersoy et al., 2019).

• 한편, JM 만 시행한 뇌졸중 환자는 통증 감소 및 신체 기능을 회복하는데 한계가 있는 것으로 관찰되었다.

• 한의학 (TKM) 치료는 회복이 더디고 (뇌졸중 등) 완전히 치료하기 어려운 만성 질환에 자주 적용되고 있다 (Koo et al., 2009).

• TKM 치료는 경락, 근육 및 경혈을 조절하거나 환부를 자극하여 생리적 균형을 조절하

는 데 효과적인 것으로 보고되었다. 침과 뜸 등의 TKM 요법은 뇌졸중 환자의 신경을 자극하여 통증을 줄이는 데 효과적이었으며 운동 기능 회복과 일상 생활 활동 (ADL) 개선에 크게 기여했다 (Kim, 2016).

• 선행연구에 따르면 TKM 치료는 뇌졸중 환자의 통증과 우울증을 개선하는 데 효과적이다 (Choi et al., 2011). 특히 TKM 치료와 다른 치료법을 병용 한 치료가 TKM 단독 치료보다 뇌졸중 환자에게 더 효과적이라고 보고되었다 (Heo et al., 2013).

• 따라서 뇌졸중 환자의 통증, 우울증, 신체 기능 회복의 한계를 개선하기 위해서는 JM과 TKM 치료를 병행하는 것이 필요하다고 생각된다.

• 하지만, JM에 대한 연구가 지속적으로 진행되었지만 통증, 우울증 및 신체 기능에 대한 TKM 치료와 결합 된 JM의 효과를 조사하는 연구는 불충분하다.

• 이에 뇌졸중 환자는 한방 치료를 병행한 관절 가동 운동 후 통증, 우울 및 신체 기능이 개선될 것이라는 가설을 세워 한방 치료를 병행한 관절 가동 운동이 뇌졸중 환자의 통증, 우울 및 신체 기능에 미치는 효과에 대해 알아보는데 그 목적이 있다.

2. 연구 실시기관 및 주소

• 실시기관 및 실시장소 : 충남 당진시 서부로 56 (당진시보건소)

3. 예상 연구 기간 및 일정표

• 연구 소요 예상 기간: IRB 심의 승인일 ~ 3개월

• 일정표

연구내용	IRB 승인일로부터 개월 수로 연구 진행 계획 표시											
	1	2	3	4	5	6	7	8	9	10	11	12
IRB 심의의뢰 및 승인												
연구대상자 모집 및 자료수집	■											
연구 진행	■	■	■									
연구 데이터 정리 및 논문 제출			■									

4. 연구비 지원기관

• 해당 없음

5. 연구자 정보

• 연구책임자 : 이호성(단국대학교 일반대학원 운동의과학과(천), 정교수)

• 연구담당자 : 이지은(단국대학교 일반대학원 운동의과학과(천), 박사과정)

6. 연구대상자

• 연구대상자는 당진시보건소에 등록되어 있는 환자 중 연구에 자발적으로 참가를 원하는 뇌졸중 환자 69명을 선정할 것이다. 연구대상자는 한방치료 및 관절가동을 병행하였

2021.06.09

을 때의 효과를 살펴보기 위해서 한방치료를 병행한 관절 가동 그룹(JT, 23명), 관절 가동 그룹(JM, 23명) 및 대조 그룹(CON, 23명)으로 무작위 분류할 것이다.

• 선정기준 : 6개월 전에 뇌졸중 진단을 받은 자, 6개월 이상 어깨와 무릎에 통증이 있는 자, 간이 정신 상태 검사(K-MMSE) 점수가 24점 이상인 자

• 제외기준 : 종양 또는 감염 위험이 있는 자, 정형외과적인 질환 및 발목 관절의 수술 병력이 있는 자, 어깨 및 무릎관절에 정형외과적인 수술 병력이 있는 자, 선 나누기 검사(LBT)에서 길이가 6.3mm 이상인 자

• 연구절차 : 연구대상자를 한방치료를 병행한 관절 가동 그룹(JT, 23명), 관절 가동 그룹(JM, 23명) 및 대조 그룹(CON, 23명)으로 무작위 분류한 후에 사전 및 치료 12주 후에 통증, 우울 및 신체 기능을 검사할 것이다. JT는 1주일에 1회 30분간, 12주간 한방치료를 시행할 것이며, 관절가동 운동은 1주일에 2회 30분간, 12주간 시행할 것이다. JM은 1주일에 2회 30분간, 12주간 관절가동 운동만 실시할 것이며, CON은 한방치료 및 관절가동을 실시하지 않을 것이다.

7. 예상 연구대상자 수와 산출 근거

• 뇌졸중 환자 특성상 모집에 대한 한계를 극복하기 위해 각 그룹에 20명 이상 모집하고자 한다.

• 각 그룹에 23명씩 뇌졸중 환자 총 69명의 연구대상자를 직접 모집하고자 한다.

8. 연구대상자 모집

• 연구대상자 모집은 당진시보건소에서 어깨 및 무릎관절의 통증 개선을 목적으로 진행하는 뇌졸중 환자를 대상으로 이 연구 내용을 설명한 후 참여를 원하는 대상자로 모집할 것이다.

9. 연구대상자 동의

• 연구담당자는 연구대상자에게 연구의 기본 과정을 설명하고 대상자 서면 동의와 설명서 및 동의서를 제공하여 연구의 취지 내용을 충분히 설명한 후 자발적으로 참여 동의를 얻을 것이다.

10. 연구 방법

• 연구대상자의 그룹 배정은 무작위 표본 추출 설계를 사용하여 한방치료를 병행한 관절 가동 그룹(JT), 관절가동 그룹(JM) 및 대조그룹(CON)으로 분류하여 12주간 실험을 실시할 것이다.

• JT의 한방치료는 1주일에 1회 30분간, 12주간 시행할 것이다. 한의사 면허증이 있는 공중보건의가 치료를 할 것이며, 간호사 면허증이 있는 7급 간호 공무원이 한방치료 보조를 할 것이다. 한방치료는 침술 치료 및 뜸 치료를 시행할 것이며, 침술 치료는 1회용 멸균 스테인리스 바늘 (0.20mm × 30mm, 한국 동방)을 평균 15분 동안 시행할 것이며, 관통 깊이는 부위에 따라 다르며 평균 20mm 깊이로 삽입할 것이다. 뜸 치료는 미니 뜸(한국 태극)을 15분간 시행할 것이다. JT의 관절가동 운동은 1주일에 2회 30분간, 12주간 시행할 것이며, 물리치료사 면허증이 있는 연구담당자가 실시할 것이다. 관절 가동은

2~3 등급으로, 어깨 및 무릎관절의 신연 및 활주를 시행할 것이며, 어깨는 바로 누운 자세 및 엎드려 누운 자세에서 오목위팔관절(Glenohumeral joint)을 가쪽 신연, 아래쪽방향 활주, 뒤쪽방향 활주 및 앞쪽 활주를 시행할 것이며, 어깨가슴관절(Scapulothoracic joint)은 올림, 내림, 벌림, 모음, 위쪽 회전 및 아래쪽 회전을 시행할 것이다. 무릎은 바로 누운 자세 및 엎드려 누운 자세에서 정강넙다리관절(Tibiofemoral joint)을 아래쪽 신연, 뒤쪽 활주 및 앞쪽 활주를 시행할 것이며, 무릎넙다리관절(Patellofemoral joint)은 을 대상으로 아래쪽 방향 활주, 가쪽 활주 및 안쪽 활주를 시행할 것이다.

- JM의 관절가동 운동은 1주일에 2회 30분간, 12주간 시행할 것이며, 물리치료사 면허증이 있는 연구담당자가 실시할 것이다. 어깨 및 무릎관절의 관절가동 방법은 JT의 관절가동 방법과 동일하게 실시할 것이다.
- CON은 한방치료 및 관절가동 모두 시행하지 않을 것이다.
- 실험 전과 실험 12주 후에 통증, 우울 및 신체 기능에 대한 검사를 실시할 것이며, 통증에 대한 검사는 100mm 표식이 있는 VAS 척도를 이용하여 시각사상척도(VAS)를 측정할 것이며, 어깨의 통증 및 장애 정도를 평가하기 위한 어깨 통증 및 장애 지수(SPADI), 무릎의 통증 및 기능 정도를 평가하기 위한 무릎 통증 및 삶의 질 척도(KWOMAC)를 평가할 것이다. 우울에 대한 검사는 자기 보고형 우울 평가 도구를 이용하여 우울증척도(CES-D)를 측정할 것이며, 정서적, 인지적, 동기적, 생리적인 우울정도를 평가할 수 있는 벡의 우울 척도(BDI)를 측정할 것이다. 신체 기능에 대한 검사는 고니오미터(Goniometer, Saehan corporation, Korea)를 사용하여 어깨 관절과 무릎 관절의 관절가동범위(ROM)를 측정할 것이며, 관절가동범위는 능동적으로 어깨 관절의 굽힘, 폄, 벌림, 모음 및 무릎 관절의 굽힘을 3회 반복 측정할 것이다. 보행 속도를 평가하기 위해서 10미터 보행 속도(10MWT)검사를 통해 14m 거리를 독립적으로 걷도록 한 후 중간 지점 10m 이동한 시간을 초(sec)단위로 3번 반복 측정할 것이다. 전반적인 보행의 기능을 평가하기 위해 기능적 보행 평가(FGA)를 통해 보행 속도 변경, 보행시 머리 회전, 계단 오르내리기, 장애물 넘기 보행 등 전반적인 보행의 기능을 측정할 것이다. 뇌졸중 환자의 팔의 전반적인 상태를 평가하기 위해 상지 기능 검사(MFT)를 실시할 것이며, 독립적인 일상생활의 능력을 평가하기 위해서 일상생활능력(ADL) 및 도구적 일상생활능력(IADL)을 평가할 것이다.

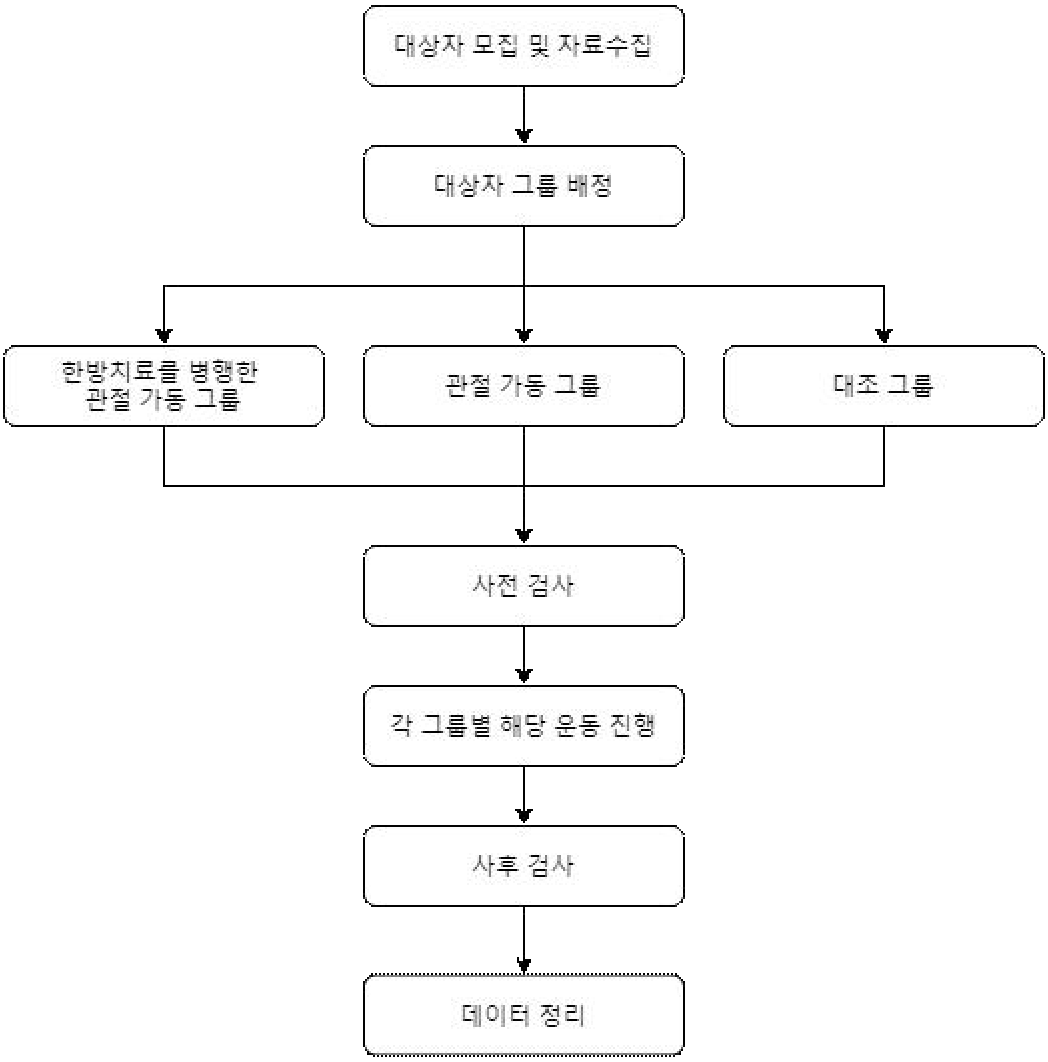

11. 효과 평가 기준 및 방법

• 한방치료를 병행한 관절가동 그룹과 관절가동 그룹의 통증, 우울 및 신체기능의 변화를 비교할 것이다.

12. 안전성 평가 기준 및 평가 방법

• VAS는 100mm 표식이 있는 척도를 이용하여 통증이 없는 상태를 0, 극심한 통증을 100으로 구분하여 대상자가 스스로 기록한 후 측정할 것이다(Wagner, 2007).
• SPADI는 어깨의 통증 및 장애 정도를 평가하기 위한 평가 도구로(Roach et al., 1991), 총 13개의 평가 항목은 0~10점 척도도 이루어져 있다. 총점 130점 중에 100점 이상일 경우 어깨의 상태가 매우 나쁜 것으로 평가할 것이다(Angst et al., 2007).
• KWOMAC은 무릎의 통증 및 기능 정도를 평가하기 위한 평가 도구로 WOMAC (Bellamy, 2000)을 한국판으로 수정된 것이다(Bae SC, 2001). 총 24개의 평가 항목은 0~4점 척도로 이루어져 있으며, 총점은 96점으로 점수가 높을수록 무릎의 상태가 매우 나쁜 것으로 평가할 것이다(Bae SC, 2001).

• CES-D는 자기 보고형 우울 평가 도구로(Radloff, 1977), 총 20개의 평가 항목으로 0~3점 척도로 이루어져 있다. 총점은 60점 중에 21점 이상일 경우 높을수록 우울한 상태인 것으로 평가할 것이다(Radloff, 1977).

• BDI는 정서적, 인지적, 동기적, 생리적인 우울정도를 평가할 수 있는 도구로(Beck, 1961), 한국판으로 번역된 것을 사용할 것이다(Lee, 1993). 총 21개의 평가 항목은 0~3점 척도로 이루어져 있으며, 총점은 63점으로 점수가 높을수록 우울 정도가 심각한 것으로 평가할 것이다(Lee, 1993).

• ROM은 고니오미터(Goniometer, Saehan corporation, Korea)를 사용하여 어깨 관절과 무릎 관절의 관절가동범위를 측정할 것이며, 능동으로 어깨 관절의 굽힘, 폄, 벌림, 모음 및 무릎 관절의 굽힘을 3회 반복 측정하여 평균값으로 평가할 것이다(Riddle, 1987).

• 10MWT는 14m 거리를 독립적으로 걷도록 한 후 중간 지점 10m 이동한 시간을 초(sec)단위로 3번 반복 측정하여 평균값으로 평가할 것이다(Steffen, Hacker, & Mollinger, 2002).

• FGA는 Wrisley(2004)가 개발한 보행 평가를 사용하여 기능적 보행 평가를 실시할 것이며, 총 10개의 항목으로, 0~3점 척도이며 총 30점으로 구성되어 있다. 구체적인 항목은 평범한 지면에서 보행하기, 보행 속도를 변경하기, 보행하면서 옆으로 머리를 돌리기, 보행하면서 상하로 머리를 움직이기, 보행하다가 한 발을 축으로 해서 돌기, 장애물 위를 지나 걷기, 좁은 기저면에서 걷기, 눈을 감고 걷기, 뒤로 걷기, 계단 오르내리기이다(Wrisley, 2004).

• MFT는 뇌졸중 환자의 팔의 전반적인 상태를 평가하기 위해 개발된 검사 도구이다. 총 8항목으로 구성되어 있으며, 하위 항목 검사가 가능하면 1점, 검사 불가능이면 0점으로 측정하여 총점은 32점이다(Miyamoto, 2009).

• ADL 및 IADL은 Won et al(2002)에 의해 개발된 도구로 ADL은 옷 입기, 세수하기, 목욕하기, 식사하기, 이동, 화장실 사용, 대소변 조절에 대한 총 7개문항으로, 1~3점 척도로 총 21점으로 구성되어 있다. IADL은 몸단장, 집안일, 식사준비, 빨래하기, 근거리 외출, 교통수단 이용, 물건 사기, 금전 관리, 전화 사용, 약 챙겨 먹기 등 총 10개 문항으로, 1~3점 척도로 총 30으로 구성되어 있다. 점수가 낮을수록 의존성이 낮고 독립적으로 일상생활이 가능한 것으로 평가할 것이다(Won et al., 2002).

13. 자료 분석과 통계적 방법

• 자료처리방법은 공분산 분석(ANCOVA)을 실시할 것이며, 중재 후 그룹 내 차이는 t-검정으로 분석할 것이며, 그룹 간의 차이는 Tukey의 사후 검정에 따른 다중비교를 실시할 것이다. 통계적 유의수준은 α=.05로 설정할 것이다.

14. 예측되는 위험/불편요소 및 대처방안

• 한방치료 중 침 치료를 시행한 후에 미세한 감염이 발생하는 것을 방지하기 위해 치료 후 해당부위에 일회용 반창고를 부착하도록 할 것이며, 침 치료 후 대상자가 해당부위에 통증 및 뻐근함을 느낄 경우 보건소 내에서 한의사 및 간호사의 판단을 통해 회복을 위한 처치를 시행할 것이다,

• 한방치료 중 뜸 치료를 시행한 후에 화상이 발생하는 것을 예방하기 위해 미니 뜸을

사용하여 간접 뜸 치료를 시행할 것이며, 뜸 치료 후 해당부위에 화상 흔적 및 물집이 발생할 경우 보건소 내에서 한의사 및 간호사의 판단을 통해 응급처치를 시행하고 손상에 대한 치료를 시행할 것이다.

• 관절 가동 운동시 극심한 통증이 발생할 경우 물리치료사인 연구담당자(이지은, 박사과정)을 통해 치료적 운동 및 통증에 대한 해당치료를 시행할 것이다.

• 예측하지 못한 부작용 및 불편감을 관리하기 위해 연구담당자(이지은, 박사과정)를 통한 모니터링을 시행할 것이다.

• 본 연구 수행 중 응급상황이 발생시 비용 부담자는 연구담당자(이지은, 박사과정)로 할 것이다.

• 최근 발생하고 있는 COVID19를 예방하기 위하여 본 실험과 관련된 연구대상자 및 연구자는 연구 기간동안 실험 전과 후에 열체크 및 소독을 실시할 것이며, 실험은 치료자와 대상자 1:1로 실시할 것이다. 또한, 연구기간동안 연구대상자 및 연구자에게서 COVID19 확진자가 발생할 경우 연구를 중단할 것이다.

15. 연구대상자가 얻을 수 있는 이익

• 한방치료를 병행한 관절가동 후에 뇌졸중 환자의 통증 감소 및 관절가동범위가 개선됨으로써 일상생활을 수행하는데 이익이 있을 것으로 생각된다.

• 3개월동안 총 24회의 연구에 참여하는 연구대상자들의 교통비에 대한 적절한 보상을 위해서 연구대상자 1인당 1개월에 10,000원으로 책정하여 3개월 후에 지역상품권 30,000원을 지급할 것이다.

16. 연구의 윤리성 확보를 위한 방안

• 연구대상자의 개인 정보는 연구와 관련한 정보 외에는 수집하지 않을 것이며, 수집한 자료는 연구 기간에만 이용하게 될 것이다.

• 연구 종료 후 연구와 관련한 자료를 연구담당자(이지은, 박사과정)가 연구 종료 시점으로부터 3년간 보관할 것이다.

• 연구 기간동안 수집된 중도철회 및 중도탈락자의 자료는 본 연구와 관련한 자료이므로 연구 종료 시점으로부터 3년간 보관할 것이다.

17. 참고문헌

• Adey-Wakeling, Z., Arima, H., Crotty, M., Leyden, J., Kleinig, T., Anderson, C. S., Newbury, J., & Collaborative, S. S. (2015). Incidence and associations of hemiplegic shoulder pain poststroke: prospective population-based study. Archives of physical medicine and rehabilitation, 96(2), 241-247.

• Angst, F., Goldhahn, J., Pap, G., Mannion, A., Roach, K., & Siebertz, D. (2007). Cross-cultural adaptation, reliability and validity of the German Shoulder Pain and Disability Index (SPADI). Rheumatology (Oxford), 46(1), 87-92.

• Bae, S. C., Lee, H. S., Yun, H. R., Kim, T. H., Yoo, D. H., & Kim, S. Y. (2001). Cross-cultural adaptation and validation of Korean Western Ontario and McMaster Universities (WOMAC) and Lequesne osteoarthritis indices for clinical research.

Osteoarthritis and cartilage, 9(8), 746-750.
• Baumann, M., Le Bihan, E., Chau, K., & Chau, N. (2014). Associations between quality of life and socioeconomic factors, functional impairments and dissatisfaction with received information and home-care services among survivors living at home two years after stroke onset. BMC neurology, 14(1), 92.
• Beck, A. T., Ward, C. H., Mendelson, M., Mock, J., & Erbaugh, J. (1961). An inventory for measuring depression. Archives of general psychiatry, 4(6), 561-571.
• Bellamy, N. (2000). WOMAC Osteoarthritis Index: User Guide IV. WOMAC. Queensland, Australia.
• Carolyn, K., & Colby, L. (2002). Therapeutic exercise foundations and techniques. Book promotion & service Ltd, 591-677.
• Choi, J. H., Kim, L. H., Yun, J. M., & Moon, B. S. (2011). Study of Clinical Research Acupuncture Treatment on Post-stroke Depression. Journal of Physiology & Pathology in Korean Medicine, 25(6), 1119-1128.
• Dobkin, B. H. (2005). Rehabilitation after stroke. New England Journal of Medicine, 352(16), 1677-1684.
• Ersoy, U., Kocak, U. Z., Unuvar, E., & Unver, B. (2019). The Acute Effect of Talocrural Joint Mobilization on Dorsiflexor Muscle Strength in Healthy Individuals: A Randomized Controlled Single-Blind Study. Journal of sport rehabilitation, 28(6), 601-605.
• Flynn, R. W. V., MacWalter, R. S. M., & Doney, A. S. F. (2008). The cost of cerebral ischaemia. Neuropharmacology, 55(3), 250-256.
• Godges, J. J., Mattson-Bell, M., Thorpe, D., & Shah, D. (2003). The immediate effects of soft tissue mobilization with proprioceptive neuromuscular facilitation on glenohumeral external rotation and overhead reach. Journal of Orthopaedic & Sports Physical Therapy, 33(12), 713-718.
• Goo, J. G., No, H. I., Hong, S. M., Kang, I. S., Lee, Y. H., & Han, D. W. (2009). Workers' attitudes about a system of collaborative hospital practice between western and traditional Korean medicine. Journal of Society of Preventive Korean Medicine, 13(2), 129-146.
• Heo, K. H., Hwang, E. H., Cho, H. W., Lee, I., Hong, J. W., Shin, Y. I., ... & Shin, B. C. (2013). An analysis of the effectiveness of stroke between east-west integrative medicine hospital and western medicine hospital by the data of brain rehabilitation registry. Journal of Korean Medicine Rehabilitation, 23(3), 117-124.
• Kaltenborn FM, Evjenth O, Kaltenborn TB, et al. Manual mobilization of the Joints The Extremities(10th ed). Yeong Mun Publishing Company. 2007.
• Kelly-Hayes, M., Beiser, A., Kase, C. S., Scaramucci, A., D’Agostino, R. B., & Wolf, P. A. (2003). The influence of gender and age on disability following ischemic stroke: the Framingham study. Journal of Stroke and Cerebrovascular Diseases, 12(3), 119-126.
• Kim, M. S., & Moon, B. S. (2016). Effect of Comprehensive Korean Medicine with

Rehabilitation in Stroke Patients: A Retrospective Study. Journal of Physiology & Pathology in Korean Medicine, 30(5), 355-359.

• Lang, C. E., Bland, M. D., Bailey, R. R., Schaefer, S. Y., & Birkenmeier, R. L. (2013). Assessment of upper extremity impairment, function, and activity after stroke: foundations for clinical decision making. Journal of Hand Therapy, 26(2), 104-115

• Lee, D. I., Kim, S. Y., Kim, K. S., Choi, D. Y., Lee, J. D., & Lee, Y. H. (2003). The evaluation of central post stroke pain. The Korean Journal of Pain, 16(2), 157-163.

• Lee, Y. H. (1993). Relations between attributional style, life events, event attribution, hopelessness and depression. Unpublished doctoral dissertation, Seoul National University, Seoul.

• Maciejasz, P., Eschweiler, J., Gerlach-Hahn, K., Jansen-Troy, A., & Leonhardt, S. (2014). A survey on robotic devices for upper limb rehabilitation. Journal of neuroengineering and rehabilitation, 11(1), 1-29.

• Marini, C., Baldassarre, M., Russo, T., De Santis, F., Sacco, S., Ciancarelli, I., & Carolei, A. (2004). Burden of first-ever ischemic stroke in the oldest old: evidence from a population-based study. Neurology, 62(1), 77-81.

• Miyamoto, S., Kondo, T., Suzukamo, Y., Michimata, A., & Izumi, S. I. (2009). Reliability and validity of the Manual Function Test in patients with stroke. American journal of physical medicine & rehabilitation, 88(3), 247-255.

• Park, Y. S. (2014). The effect of dynamic stretching and Evjenth-hamberg stretching at ankle joint on balance and gait in patients with stroke. Master's Degree. Yong-in University.

• Radloff, L. S. (1977). The CES-D scale: A self-report depression scale for research in the general population. Journal of Applied Psychological Measures, 1, 385-401.

• Roach, K. E., Budiman-Mak, E., Songsiridej, N., & Lertratanakul, Y. (1991). Development of a shoulder pain and disability index. Arthritis Care Res, 4(4), 143-149.

• Steffen, T. M., Hacker, T. A., & Mollinger, L. (2002). Age- and Gender-Related Test Performance in Community-Dwelling Elderly People: Six-Minute Walk Test, Berg Balance Scale, Timed Up & Go Test, and Gait Speeds. Physical Therapy, 82(2), 128–137.

• Vér, C., Hofgárt, G., Menyhárt, L., Kardos, L., & Csiba, L. (2015). Ankle-foot continuous passive motion device for mobilization of acute stroke patients. Open Journal of Therapy and Rehabilitation, 3(02), 23.

• Wagner, D. R., Tatsugawa, K., Parker, D., & Young, T. A. (2007). Reliability and utility of a visual analog scale for the assessment of acute mountain sickness. High altitude medicine & biology, 8(1), 27-31.

• Won, C. W., Yang, K. Y., Rho, Y. G., Kim, S. Y., Lee, E. J., Yoon, J. L., ... & Yoon, D. K. (2002). The development of Korean activities of daily living (K-ADL) and Korean instrumental activities of daily living (K-IADL) scale. J Korean Geriatr Soc, 6(2), 107.

• Wrisley, D. M., Marchetti, G. F., Kuharsky, D. K., & Whitney, S. L. (2004). Reliability, internal consistency, and validity of data obtained with the functional gait

assessment. Physical therapy, 84(10), 906-918.

• Yang, A., Wu, H. M., Tang, J. L., Xu, L., Yang, M., & Liu, G. J. (2016). Acupuncture for stroke rehabilitation. Cochrane Database of Systematic Reviews, (8).

• Yoo, C., Yong, M. H., Chung, J., & Yang, Y. (2015). Effect of computerized cognitive rehabilitation program on cognitive function and activities of living in stroke patients. Journal of Physical Therapy Science, 27(8), 2487-2489.

신 청 일	2021년 04월 24일
연구책임자	이 호 성 (인/서명)
