# Supplementary material for: Effects of joint mobilization combined with acupuncture on pain, physical function, and depression in stroke patients with chronic neuropathic pain: A randomized controlled trial
Source: PLoS One. 2023 Aug 24;18(8):e0281968. doi: 10.1371/journal.pone.0281968 (PMC10449141; doi:10.1371/journal.pone.0281968)

[Form 4-1]

# Study Protocol (for human subject research)

## Version 2.0

| | |
|---|---|
| Research title: | Effects of joint mobilization combined with Korean medicine treatment on pain, physical function, and depression in stroke patients with chronic neuropathic pain: a randomized controlled trial |

**Dankook University Graduate School**
**Department of Exercise Medicine Lee Ho-seong**

# 1. Purpose and background of the study

• Stroke is the most common cause of upper extremity (UL) movement disorders in adults (Maciejasz P, 2014), and hemiplegic shoulder pain (HSP) is a serious complication experienced by approximately 30% of survivors within 1 year (Adey-Wakeling, 2015).
• Only one-third of stroke patients recover function within the first 6 months and motor recovery is poor or insufficient (Dobkin, 2005). ). While less than 45% of stroke patients are likely to achieve full functional recovery, most present with various types of sequelae and difficulty performing activities of daily living (Kelly-Hayes et al., 2003; Marini et al. , 2004; Flynn et al., 2008).
• In addition to motor control and sensory deficits, stroke presents with common complications such as pain, spasticity, joint limitations, and skin or blood vessel damage, which are the most important problems in stroke management (Lang et al., 2013; Ver et al., 2015).
• Restoration of gait function is the main goal of rehabilitation, and most stroke patients have difficulty walking due to reduced gait speed (Yoo & Chung, 2015). Independent ambulation is an important factor in performing activities of daily living after stroke, and previous studies have shown that sensorimotor dysfunction causes range of motion (ROM) limitations and weakness of the lower extremity muscles on the paretic side, making it difficult to sit to stand and walk. difficulty performing the same functional activities (Lyn et al. al., 2006; Fujita et al., 2011).
• Therefore, in order to improve the physical function of stroke patients, it is necessary to reduce joint pain and expand the range of motion. A variety of methods designed to address these problems, help restore and improve upper and lower extremity function, and provide rehabilitation are widely used by therapists.
• Mulligan was the first to propose motion-assisted mobilization as a joint mobilization (JM) technique. Joint mobilization is an orthopedic method used for mechanical effect to relieve pain, improve movement, and treat contractures through three phases of movement and the convex-concave rule Kaltenborn et al., 2007).
• Joint mobility includes traction, compression and gliding and is divided into grades I-III. Class I is distraction with some intensity without separation of the bonding surfaces . Grade II isolates the articular surface by stretching the periarticular tissue within the joint capsule. Grade III requires sufficient distraction or weeks to allow the joint capsule to stretch sufficiently (Kaltenborn et al., 2007). According to the convex and concave rule, sliding on a convex joint surface means sliding in the direction opposite to the direction of bone movement. Sliding on a concave joint surface is to implement sliding in the direction opposite to the direction of bone movement. When separating the

concave articular surfaces, they are pulled along the long axis of the bone. However, when distracting the convex articular surfaces, the articular surfaces must be separated (Kaltenborn et al., 2007).

• Joint motion has not only a mechanical effect that hinders contracture by direct movement of the joint area, but also a joint motion reflex effect that promotes the inhibition or stimulation of related muscles by promoting joint receptors (Ersoy et al., 2019).

• On the other hand, it was observed that stroke patients who received only JM had limitations in reducing pain and restoring physical functions.

• Traditional Korean medicine (TKM) treatment is frequently applied to chronic diseases that are difficult to treat completely and have slow recovery (such as stroke) (Koo et al., 2009).

• TKM treatment has been reported to be effective in regulating the physiological balance by regulating meridians, muscles and acupuncture points, or by stimulating the affected area. TKM therapy, such as acupuncture and moxibustion, was effective in reducing pain by stimulating the nerves of stroke patients and contributed greatly to the recovery of motor function and improvement of activities of daily living (ADL) (Kim, 2016).

• According to previous studies, TKM treatment is effective in improving pain and depression in stroke patients (Choi et al., 2011). In particular, it has been reported that TKM treatment combined with other therapies is more effective for stroke patients than TKM treatment alone (Heo et al., 2013).

• Therefore, it is considered necessary to combine JM and TKM treatment to improve the limitations of pain, depression, and physical function recovery in stroke patients.

• However, although research on JM continues, there are insufficient studies examining the effects of JM combined with TKM treatment on pain, depression and physical function.

• Therefore, we hypothesized that pain, depression, and physical function would be improved after joint mobilization exercise combined with oriental medicine treatment in stroke patients, and the effects of joint mobilization exercise combined with oriental medicine treatment on pain, depression, and physical function in stroke patients were investigated. Its purpose is to find out.

## 2. Research organization and address

• Implementation institution and location: 56 Seobu-ro, Dangjin-si , Chungcheongnam -do ( Dangjin-si Public Health Center)

## 3. Estimated study duration and timeline

• Expected duration of research: IRB review approval date ~ 3 months

• Schedule

| Research contents | Indicates study progress plan in number of months from date of IRB approval | | | | | | | | | | | |
|---|---|---|---|---|---|---|---|---|---|---|---|---|
| | 1 | 2 | 3 | 4 | 5 | 6 | 7 | 8 | 9 | 10 | 11 | 12 |
| IRB review request and approval | | | | | | | | | | | | |
| Subjects and data collection | | | | | | | | | | | | |
| Research progress | | | | | | | | | | | | |
| Data and submit papers | | | | | | | | | | | | |

## 4. Research fund support organization

• Not applicable

## 5. Researcher information

• Principal Investigator: Lee Ho-seong (Department of Exercise Medicine, Dankook University Graduate School, Full Professor)
• Researcher: Lee Ji-eun (Dankook University Graduate School of Exercise Medicine (Cheon), doctoral course)

## 6. Research subjects

• Study subjects will select 69 stroke patients who voluntarily want to participate in the study among patients registered at the Dangjin City Public Health Center . In order to examine the effects of combining oriental medicine treatment and joint mobility, the study subjects were divided into joint mobilization group (JT, 23 patients), joint mobilization group (JM, 23 patients) and control group (CON, 23 patients) combined with oriental medicine treatment. ) will be randomly assigned.
• Selection Criteria: Those diagnosed with a stroke 6 months ago, those with shoulder and knee pain for more than 6 months, those with a K-MMSE score of 24 or higher
• Exclusion Criteria: Those at risk of tumor or infection, those with a history of orthopedic diseases and ankle joint surgery, those with a history of orthopedic surgery on the shoulder and knee joints, and those with a length of 6.3 in the LBT test. mm or more
• Study procedure: Subjects were randomly classified into joint mobilization group (JT, 23 patients), joint mobilization group (JM, 23 patients), and control

group (CON, 23 patients) concurrently treated with oriental medicine, and 12 weeks before and after treatment Pain, depression, and physical function will be tested later. JT will perform oriental medicine treatment for 30 minutes once a week for 12 weeks, and joint mobilization exercise will be performed twice a week for 30 minutes for 12 weeks. JM will perform only joint mobilization exercises for 30 minutes twice a week for 12 weeks, and CON will not perform oriental medicine treatment and joint mobilization.

## 7. Estimated number of research subjects and calculation basis

• We plan to recruit more than 20 people in each group to overcome the limitation of recruitment due to the characteristics of stroke patients.

• A total of 69 subjects of stroke, 23 in each group, were recruited directly.

## 8. Recruitment of research subjects

• Recruitment of research subjects will be recruited after explaining the contents of this study to stroke patients who are progressing for the purpose of improving shoulder and knee joint pain at the Dangjin City Public Health Center.

## 9. Consent of research subjects

• The person in charge of the research will explain the basic process of the research to the research subject, provide the subject's written consent, explanation, and consent form to sufficiently explain the purpose of the research, and then voluntarily obtain consent to participate.

## 10. Research methods

• The group assignment of study subjects will be conducted for 12 weeks by classifying them into joint mobility group (JT), joint mobility group (JM), and control group (CON) combined with oriental medicine treatment using random sampling design.

• JT's herbal treatment will be performed once a week for 30 minutes for 12 weeks. A public health doctor with an oriental doctor's license will treat, and a 7th grade nursing official with a nurse's license will assist in oriental treatment. Acupuncture treatment and moxibustion treatment will be performed for oriental medicine treatment, and acupuncture treatment will be performed with a disposable sterilized stainless steel needle (0.20mm × 30mm, Dongbang, Korea) for an average of 15 minutes. will insert Moxibustion treatment will be performed for 15 minutes with mini-moxibustion (Korean Taegeuk). JT's joint mobilization exercise will be performed twice a week for 30 minutes for 12 weeks, and it will be conducted by a researcher with a physical therapist

license. Joint mobility is grade 2-3, distraction and gliding of the shoulder and knee joints will be performed, and the shoulder will move the glenohumeral joint laterally in the supine and supine position . Distraction , downward glide, posterior glide, and anterior glide will be performed, and the scapulothoracic joint will be raised, lowered, abducted, lowered, rotated upward, and rotated downward. For the knee , downward distraction , posterior glide, and anterior glide of the tibiofemoral joint will be performed in the supine and prone position, and downward glide and lateral glide for the Patellofemoral joint . and inner slides.

• JM's joint mobilization exercise will be performed twice a week for 30 minutes for 12 weeks, and will be conducted by a researcher with a physical therapist license. The joint mobilization method of the shoulder and knee joints will be performed in the same way as the joint mobilization method of JT.

• CON will not perform both herbal treatment and joint mobilization.

• Tests for pain, depression, and physical function will be conducted before and after 12 weeks of the experiment, and the test for pain will be measured using a VAS scale with a 100mm mark on the visual mapping scale (VAS). Shoulder pain and The Shoulder Pain and Disability Index (SPADI) to assess the degree of disability and the Knee Pain and Quality of Life Scale (KWOMAC) to assess the degree of knee pain and function will be assessed. The depression test will measure the Depression Scale (CES-D) using the self-report depression assessment tool, and the Beck Depression Scale (BDI), which can evaluate the emotional, cognitive, motivational, and physiological levels of depression. will measure The physical function test will measure the range of motion (ROM) of the shoulder joint and knee joint using a goniometer (Goniometer, Saehan corporation, Korea). Abduction, deflection and flexion of the knee joint will be measured three times. In order to evaluate walking speed, the 10-meter walking speed (10MWT) test will be used to independently walk a distance of 14 m, and then the time to move 10 m to the intermediate point will be measured three times in seconds (sec). To evaluate the overall gait function, the functional gait assessment (FGA) will measure the overall gait function, such as change in gait speed, head rotation during walking , going up and down stairs, and gait over obstacles. An upper extremity function test (MFT) will be performed to evaluate the overall condition of the arm of stroke patients, and the ability for daily living (ADL) and instrumental daily living (IADL) will be assessed to evaluate the ability for independent daily living.

[Form 4-1]

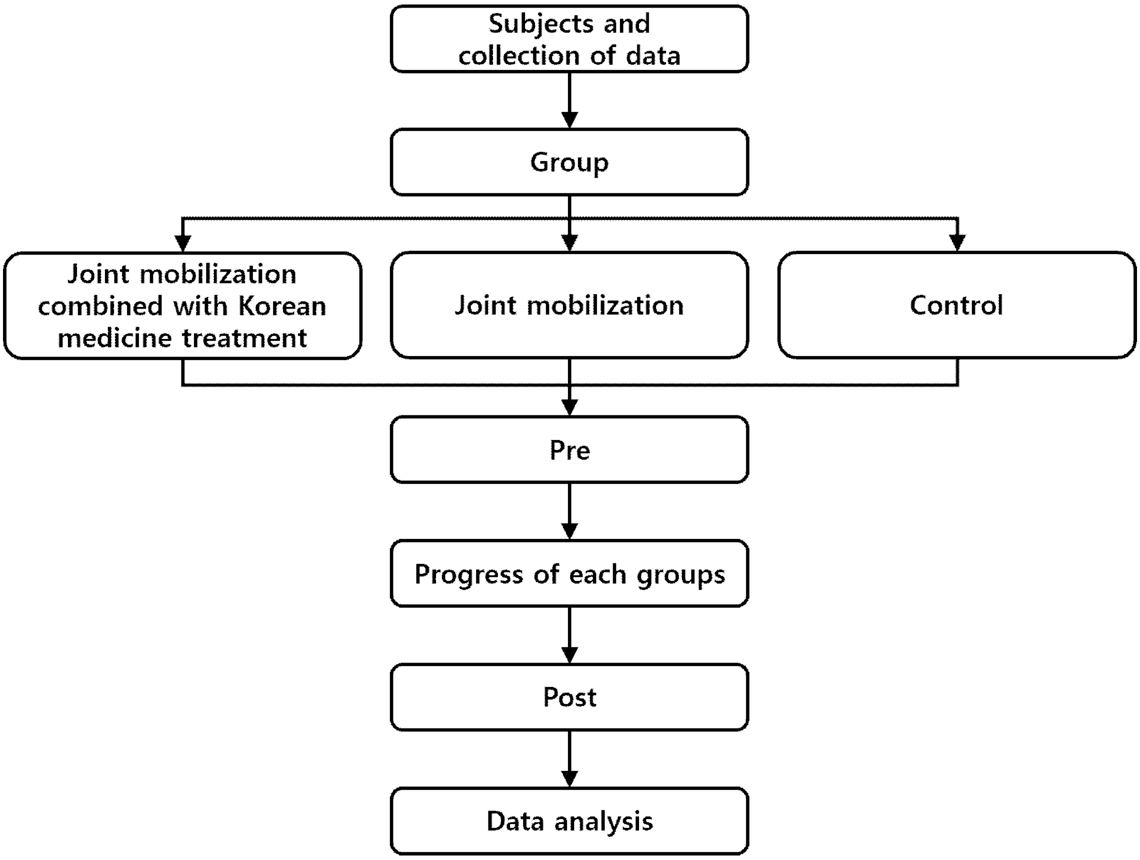


## 11. Effect Evaluation Criteria and Methods

• Changes in pain, depression, and physical function of the joint mobility group and the joint mobility group combined with oriental treatment will be compared.

## 12. Safety evaluation criteria and evaluation method

• VAS will be measured after self-recording by classifying no pain as 0 and extreme pain as 100 using a scale with a 100mm mark (Wagner, 2007).

• SPADI is an evaluation tool to evaluate the degree of pain and disability of the shoulder (Roach et al., 1991), and a total of 13 evaluation items are also composed of a 0-10 point scale. If the total score exceeds 100 out of 130, the condition of the shoulder will be evaluated as very bad (Angst et al., 2007).

• KWOMAC is a Korean version of WOMAC (Bellamy, 2000) modified as an evaluation tool to evaluate knee pain and functional level (Bae SC, 2001). A total of 24 evaluation items are composed of a 0 to 4 point scale, and the total score is 96 points, and the higher the score, the worse the condition of the knee is evaluated (Bae SC, 2001).

• CES-D is a self-report depression assessment tool (Radloff , 1977), and consists of a total of 20 evaluation items on a scale of 0 to 3 points. If the total score is 21 or more out of 60 points, the higher the total score, the more

depressed it will be evaluated (Radloff , 1977).
• The BDI is a tool that can evaluate the emotional, cognitive, motivational, and physiological levels of depression (Beck, 1961), and the Korean version will be used (Lee, 1993). A total of 21 evaluation items are composed of a 0 to 3 point scale, and the total score is 63 points, and the higher the score, the more severe the degree of depression will be evaluated (Lee, 1993).
• ROM will measure the range of motion of the shoulder joint and knee joint using a goniometer (Goniometer, Saehan corporation, Korea), and actively measure shoulder joint flexion, extension, abduction, deflection, and knee joint flexion. It will be evaluated as an average value by repeated measurements (Riddle, 1987).
• The 10MWT will be evaluated as an average value by measuring the time to move 10m to the middle point 3 times in seconds after having them walk independently for a distance of 14m (Steffen, Hacker, & Mollinger , 2002).
• FGA will conduct functional gait evaluation using the gait evaluation developed by Wrisley (2004), with a total of 10 items, on a scale of 0 to 3, with a total of 30 points. Specific items include walking on plain ground, changing walking speed, turning the head sideways while walking, moving the head up and down while walking, turning on one foot while walking, walking over obstacles, and narrow base surfaces. walking, walking with eyes closed, backward walking, and going up and down stairs (Wrisley , 2004).
• MFT is a screening tool developed to evaluate the overall condition of the arm in stroke patients. It consists of a total of 8 items, and if the sub-item can be tested, 1 point is obtained, and if the test is not possible, 0 points are measured, and the total score is 32 points (Miyamoto, 2009).
• ADL and IADL are tools developed by Won et al (2002). The ADL is a total of 7 questions about dressing, washing, bathing, eating, moving, using the toilet, and control of bowel and bladder, 1 to 3 points. The scale consists of a total of 21 points. The IADL consists of a total of 30 items on a 1-3 point scale with a total of 10 items, including grooming, housework, meal preparation, laundry, going out a short distance, using transportation, shopping for goods, money management, using the phone, and taking medicine. . The lower the score, the lower the dependency and the ability to live independently (Won et al., 2002).

## 13. Data Analysis and Statistical Methods

• As for the data processing method, analysis of covariance (ANCOVA) will be conducted, and differences within groups after intervention will be analyzed by t-test, and differences between groups will be subjected to multiple comparisons according to Tukey's post-hoc test. The statistical significance level will be set at $\alpha$=.05.

## 14. Anticipated risks/inconvenience factors and countermeasures

• To prevent microscopic infection after acupuncture treatment during oriental medicine treatment, a disposable bandage will be attached to the affected area after treatment, and if the subject feels pain or stiffness in the affected area after acupuncture treatment, an oriental doctor will be and, at the discretion of the nurse, treatment for recovery will be administered.
• Indirect moxibustion treatment will be performed using mini-moxibustion to prevent burns after moxibustion treatment among oriental medicine treatment. At the discretion of the patient, first aid will be administered and treatment for injuries will be administered.
• If severe pain occurs during joint mobilization exercise, therapeutic exercise and corresponding treatment for pain will be performed by a physical therapist (Lee Ji-eun, Ph.D).
• In order to manage unexpected side effects and discomfort, monitoring will be conducted through the researcher (Lee Ji-eun, Ph.D).
• In the event of an emergency during the conduct of this research, the person in charge of the study (Lee Ji-eun, Ph.D) will bear the cost.
• In order to prevent COVID19, which has recently occurred, research subjects and researchers related to this experiment will conduct thermal checks and disinfection before and after the experiment during the research period, and the experiment will be conducted 1:1 between the therapist and the subject. In addition, if a confirmed case of COVID19 occurs in a research subject or researcher during the research period, the research will be stopped.

## 15. Benefits that can be obtained by research subjects

• It is thought that there will be benefits in carrying out daily life by reducing pain and improving joint range of motion in stroke patients after joint mobility combined with oriental medicine treatment.
• In order to properly compensate for the transportation expenses of research subjects who participate in a total of 24 studies for 3 months, KRW 10,000 per research subject per month will be set, and KRW 30,000 in regional gift certificates will be provided after 3 months.

## 16. Measures to secure research ethics

• Personal information of research subjects will not be collected except for research-related information, and the collected data will be used only during the research period.
• After the completion of the study, the researcher (Lee Ji-eun, Ph.D) will keep the data related to the study for 3 years from the end of the study.
• The data of dropouts and dropouts collected during the study period will be

kept for 3 years from the end of the study as it is related to this study.

## 17. References

• Adey- Wakeling , Z., Arima, H., Crotty, M., Leyden, J., Kleinig, T., Anderson, CS, Newbury, J., & Collaborative, SS (2015). Incidence and associations of hemiplegic shoulder pain poststroke: a prospective population-based study. Archives of physical medicine and rehabilitation, 96(2), 241-247.

• Angst, F., Goldhahn , J., Pap, G., Mannion, A., Roach, K., & Siebertz , D. (2007). Cross-cultural adaptation, reliability and validity of the German Shoulder Pain and Disability Index (SPADI). Rheumatology (Oxford), 46(1), 87-92.

• Bae, SC, Lee, HS, Yun, HR, Kim, TH, Yoo , DH, & Kim, SY (2001). Cross-cultural adaptation and validation of Korean Western Ontario and McMaster Universities (WOMAC) and Lequesne osteoarthritis indices for clinical research. Osteoarthritis and cartilage, 9(8), 746-750.

• Baumann, M., Le Bihan , E., Chau, K., & Chau, N. (2014). Associations between quality of life and socioeconomic factors, functional impairments and dissatisfaction with received information and home-care services among survivors living at home two years after stroke onset. BMC neurology, 14(1), 92.

• Beck, AT, Ward, CH, Mendelson, M., Mock, J., & Erbaugh , J. (1961). An inventory for measuring depression. Archives of general psychiatry, 4(6), 561-571.

• Bellamy, N. (2000). WOMAC Osteoarthritis Index: User Guide IV. WOMAC. Queensland, Australia.

• Carolyn, K., & Colby, L. (2002). Therapeutic exercise foundations and techniques. Book promotion & service Ltd, 591-677.

• Choi, JH, Kim, LH, Yun, JM, & Moon, BS (2011). Study of Clinical Research Acupuncture Treatment on Post-stroke Depression. Journal of Physiology & Pathology in Korean Medicine, 25(6), 1119-1128.

• Dobkin , BH (2005). Rehabilitation after stroke. New England Journal of Medicine, 352(16), 1677-1684.

• Ersoy , U., Kocak , UZ, Unuvar , E., & Unver, B. (2019). The Acute Effect of Talocrural Joint Mobilization on Dorsiflexor Muscle Strength in Healthy Individuals: A Randomized Controlled Single-Blind Study. Journal of sport rehabilitation, 28(6), 601-605.

• Flynn, RWV, MacWalter , RSM, & Doney , ASF (2008). The cost of cerebral ischaemia . Neuropharmacology, 55(3), 250-256.

• Godges , JJ, Mattson-Bell, M., Thorpe, D., & Shah, D. (2003). The immediate effects of soft tissue mobilization with proprioceptive neuromuscular facilitation on glenohumeral external rotation and overhead reach. Journal of Orthopedic & Sports Physical Therapy, 33(12), 713-718.

• Goo, JG, No, HI, Hong, SM, Kang, IS, Lee, YH, & Han, DW (2009). Workers'

attitudes about a system of collaborative hospital practice between western and traditional Korean medicine. Journal of Society of Preventive Korean Medicine, 13(2), 129-146.
• Heo , KH, Hwang, EH, Cho, HW, Lee, I., Hong, JW, Shin, YI, ... & Shin, BC (2013). An analysis of the effectiveness of stroke between east-west integrative medicine hospital and western medicine hospital by the data of brain rehabilitation registry. Journal of Korean Medicine Rehabilitation, 23(3), 117-124.
• Kaltenborn FM, Evjenth O, Kaltenborn TB, et al. Manual mobilization of the Joints The Extremities ( 10th ed). Yeong Mun Publishing Company. 2007.
• Kelly-Hayes, M., Beiser , A., Kase , CS, Scaramucci, A., D'Agostino, RB, & Wolf, PA (2003). The influence of gender and age on disability following ischemic stroke: the Framingham study. Journal of Stroke and Cerebrovascular Diseases, 12(3), 119-126.
• Kim, MS, & Moon, BS (2016). Effect of Comprehensive Korean Medicine with Rehabilitation in Stroke Patients: A Retrospective Study. Journal of Physiology & Pathology in Korean Medicine, 30(5), 355-359.
• Lang, CE, Bland, MD, Bailey, RR, Schaefer, SY, & Birkenmeier , RL (2013). Assessment of upper extremity impairment, function, and activity after stroke: foundations for clinical decision making. Journal of Hand Therapy, 26(2), 104-115
• Lee, DI, Kim, SY, Kim, KS, Choi, DY, Lee, JD, & Lee, YH (2003). The evaluation of central post stroke pain. The Korean Journal of Pain, 16(2), 157-163.
• Lee, YH (1993). Relations between attributional style, life events, event attribution, hopelessness and depression. Unpublished doctoral dissertation, Seoul National University, Seoul.
• Maciejasz , P., Eschweiler, J., Gerlach-Hahn, K., Jansen-Troy, A., & Leonhardt, S. (2014). A survey on robotic devices for upper limb rehabilitation. Journal of neuroengineering and rehabilitation, 11(1), 1-29.
• Marini, C., Baldassarre , M., Russo, T., De Santis , F., Sacco, S., Ciancarelli , I., & Carolei , A. (2004). Burden of first-ever ischemic stroke in the oldest old: evidence from a population-based study. Neurology, 62(1), 77-81.
• Miyamoto, S., Kondo, T., Suzukamo , Y., Michimata , A., & Izumi, SI (2009). Reliability and validity of the Manual Function Test in patients with stroke. American journal of physical medicine & rehabilitation, 88(3), 247-255.
• Park, YS (2014). The effect of dynamic stretching and Evjenth-hamberg stretching at ankle joint on balance and gait in patients with stroke. Master's Degree. Yong-in University .
• Radloff , LS (1977). The CES-D scale: A self-report depression scale for research in the general population. Journal of Applied Psychological Measures, 1, 385-401.

• Roach, KE, Budiman- Mak , E., Songsiridej , N., & Lertratanakul , Y. (1991). Development of a shoulder pain and disability index. Arthritis Care Res, 4(4), 143-149.

• Steffen, TM, Hacker, TA, & Mollinger , L. (2002). Age- and Gender-Related Test Performance in Community-Dwelling Elderly People: Six-Minute Walk Test, Berg Balance Scale, Timed Up & Go Test, and Gait Speeds. Physical Therapy, 82(2), 128-137.

• Vér , C., Hofgárt , G., Menyhárt , L., Kardos , L., & Csiba , L. (2015). Ankle-foot continuous passive motion device for mobilization of acute stroke patients. Open Journal of Therapy and Rehabilitation, 3(02), 23.

• Wagner, DR, Tatsugawa , K., Parker, D., & Young, TA (2007). Reliability and utility of a visual analog scale for the assessment of acute mountain sickness. High altitude medicine & biology, 8(1), 27-31.

• Won, CW, Yang, KY, Rho, YG, Kim, SY, Lee, EJ, Yoon, JL, ... & Yoon, DK (2002). The development of Korean activities of daily living (K-ADL) and Korean instrumental activities of daily living (K-IADL) scale. J Korean Geriatr Soc, 6(2), 107.

• Wrisley , DM, Marchetti, GF, Kuharsky , DK, & Whitney, SL (2004). Reliability, internal consistency, and validity of data obtained with the functional gait assessment. Physical therapy, 84(10), 906-918.

• Yang, A., Wu, HM, Tang, JL, Xu, L., Yang, M., & Liu, GJ (2016). Acupuncture for stroke rehabilitation. Cochrane Database of Systematic Reviews, (8).

• Yoo , C., Yong, MH, Chung, J., & Yang, Y. (2015). Effect of computerized cognitive rehabilitation program on cognitive function and activities of living in stroke patients. Journal of Physical Therapy Science, 27(8), 2487-2489.


Application date April 24, 2021

Research director Ho-Seung Lee (Seal/Signature)

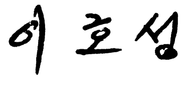

Supplement: S2 File — (PDF) [file pone.0281968.s003.pdf]
