# Supplementary material for: Effects of joint mobilization combined with acupuncture on pain, physical function, and depression in stroke patients with chronic neuropathic pain: A randomized controlled trial
Source: PLoS One. 2023 Aug 24;18(8):e0281968. doi: 10.1371/journal.pone.0281968 (PMC10449141; doi:10.1371/journal.pone.0281968)
Supplement: Attachment — Submitted filename: Response to Reviewers.docx [file pone.0281968.s004.docx]

**Response to Reviewers**

PONE-D-23-03014

Effects of joint mobilization combined with acupuncture on pain, physical function, and depression in stroke patients with chronic neuropathic pain: a randomized controlled trial

PLOS ONE

**Editor:**
Thank you for submitting your manuscript to PLOS ONE. After careful consideration, we feel that it has merit but does not fully meet PLOS ONE’s publication criteria as it currently stands. Therefore, we invite you to submit a revised version of the manuscript that addresses the points raised during the review process.

As a scientific study of acupuncture and moxibustion, the most fatal problem in this paper is, as pointed out by reviewer 2, that acupuncture and moxibustion MUST provide consistent basic acupoints, and show and write the name and location of the acupoints, e.g. based on "WHO Standard Acupuncture Point Locations ( <https://apps.who.int/iris/handle/10665/353407>)". Also, if possible, the authors could provide specific images and videos of joint mobilization techniques.
Response: Thank you very much for allowing us to revise our manuscript. We have carefully considered the comments and tried our best to address every one of them. We hope the changes and responses meet the expectation of PLOS ONE and the manuscript is now suitable for publication.

**Reviewer #1:**
This manuscript reports the results of a study in post-stroke patients of two competing treatments and a control treatment. The manuscript is generally well-written and the study appears to be well-performed.
Response: We appreciate your precious time in reviewing our paper and providing valuable comments.

Line 49: Change to "post-stroke".
Response: We have changed on Page 3, Line 49.

Line 210: Delete "statistically".
Response: We have deleted it.

Please provide more detail on the methodology used to perform the two-way analysis with repeated measures.

Response: We appreciate your thoughtful comment about our analysis. We have added some sentences regarding statistics on Page 13, Line 237-241.

Was this implemented within the linear mixed model framework? If so please describe the model in terms of fixed effects, G-side terms, and R-side terms (if any). Or was this implemented within the older framework using Greenhouse-Geisser or Huynh-Feldt correction? If so please describe the methodology used to assess sphericity. Or was this implemented using the older framework using a multivariate analysis of variance? If so, please describe the tests used.
Response: We took advantage of 2-way ANOVA with repeated measures instead of using a linear mixed model framework or specific sphericity correction methods in our analysis. We have added some sentences regarding this on Page 13, Line 237-241.

Line 228: For this and other tables, please provide the analysis of variance p-values for the terms in the model --- or at least consider providing these as supplementary information. Please footnote the table to provide the statistical tests or multiple comparison methods used.
Response: We have added these, accordingly.

Note that pairwise comparison methods that control the experimentwise error rate may be used regardless of the analysis of variance results. However, it is best to assess the data for interaction effects before assuming the main effects structure.
Response: Thank you for the valuable comment. We added the results for interaction effects to the manuscript on pages 14-19.

Line 235: Change "between" to "among". Or clarify that this "between" refers to pairwise comparisons.
Response: We have corrected it.

Line 314: Change to "The majority... were older farmers...".

Response: We have corrected it. Thank you.

**Reviewer #2:**

1. In my opinion, a reasonable randomized controlled trial should provide a formula for calculating sample size, but this paper does not.

Response: Thank you very much for the comment. We have added it on Page 6, Line 109-115.

2. Personally, I think acupuncture and moxibustion should provide consistent basic acupoints. This paper does not clearly give the name and location of the acupoints taken.

Response: Thank you very much for the valuable comment. We added the information on Page 9, Line 168-172 and revised the manuscript accordingly.

**Reviewer #3:**

Abstract: To investigate....(AC) for THE treatment OF pain, ... (missing words)
Response: Thank you very much for the comment. We have corrected it.

Introduction line 44 "Stroke is one of (how many? what percentage)?
Response: Thank you very much for the comment. We have changed it.

This sentence is confusing (line 73): "The convex-concave rule states that gliding on a convex joint surface should be done the opposite way the bone moves, and gliding on a concave joint surface should be done the opposite way." Not sure why both state "opposite way."
Response: We are sorry for the confusion. We have corrected the sentence. Thank you.

Reference for line 80?
Response: We have corrected the reference. Thank you.

Table 1: the JM+AC group were younger, with less time since onset. Although not a significant p value, these differences need to be addressed.
Response: We appreciate your comment. We are aware of the limitations in interpreting the differences in age and time since onset found in the JM+AC group. We are afraid that it may be difficult to generalize the results to other populations due to the small sample size in our study. We accordingly made changes to the limitations of the manuscript on Page 23, Line 351-357.

Line 150: Was there standard acupuncture points used? Or was the AC treatment individualized?

Response: Thank you very much for the comment. We added the information on Page 9, Line 168-172 and revised the manuscript accordingly.
